# Health technology assessment of new drugs for rare disorders in Canada: impact of disease prevalence and cost

**DOI:** 10.1186/s13023-017-0611-7

**Published:** 2017-03-23

**Authors:** Nigel S. B. Rawson

**Affiliations:** 1 Eastlake Research Group, Oakville, ON Canada; 2 Canadian Health Policy Institute, Toronto, ON Canada; 3 Fraser Institute, Vancouver, BC Canada

**Keywords:** Rare diseases, Orphan drugs, Health technology assessment, Canada

## Abstract

**Background:**

Authors from the Canadian Agency for Drugs and Technologies in Health (CADTH) presented an analysis of submissions to the Common Drug Review (CDR) between 2004 and February 3, 2016 for drugs for rare disorders (disorders with a prevalence of <50 per 100,000).

**Objective:**

The aim of this analysis was to examine the same CDR submissions to evaluate whether the negative reimbursement recommendation rate, clinical evidence of efficacy and statements concerning the drug’s cost in the CDR reports varied with the prevalence of the disorder treated by the drug grouped into three decreasing categories: <50 to >10, ≤10 to >1, and ≤1 per 100,000.

**Results:**

As the prevalence of the treated disorder decreased, the median daily cost of the drug, the negative recommendation rate and the proportion of submissions with statements in the CDR reports highlighting the cost of the drug increased, while the proportion of submissions with acceptable evidence of clinical efficacy decreased. Moreover, although the CADTH authors reported that only two submissions received a negative recommendation due to a “lack of cost-effectiveness/high cost,” high cost was mentioned in the CDR reports of 15 drugs with negative recommendations, all for disorders with a prevalence of ≤10 per 100,000.

**Conclusions:**

The aggregated analysis of CDR submissions for drugs for disorders with wide ranging prevalence rates concealed information of concern to patients. The negative reimbursement recommendation rate and the significance of cost in the CDR assessments increased as the prevalence of the treated disorder decreased. Since 2012, the manner in which high cost drugs for rare disorders have been dealt with by the CDR has changed. Cost has ceased to be a factor in negative recommendations but is included in criteria accompanying positive recommendations. This trend is associated with the integration of the CDR process with the system for price negotiation between public drug plans and pharmaceutical companies.

## Background

In Canada, the health technology assessment of non-oncology drugs for consideration for reimbursement in public drug plans is performed by the Canadian Agency for Drugs and Technologies in Health (CADTH) through its Common Drug Review (CDR) process. The CDR’s approach to evaluating the value of drugs for rare disorders (DRDs) has long been criticized [1–5] as being heavily weighted towards negative reimbursement recommendations that have led to inequitable access to DRDs across Canada. Proposals for a separate, more broad-based process for these drugs have been rejected by CADTH [6].

Employees of CADTH recently reported on an analysis of submissions for DRDs to the CDR between 2004 and February 3, 2016 [7]. They used Health Canada’s definition of a rare disorder, i.e. a prevalence of <50 per 100,000 individuals [8]. The CADTH authors found that more than half of the submissions received a positive reimbursement recommendation and that the main reason for a negative recommendation was a lack of clinical efficacy [7]. They also reported that a “lack of cost-effectiveness/high cost” was the reason for a negative recommendation in only two submissions and that “high cost was not a reason for a negative reimbursement recommendation in any recommendation after October 2012.”

The objective of the present analysis was to examine the same CDR submissions to assess whether the negative recommendation rate, level of clinical evidence and statements in the CDR reports regarding the drug’s cost varied with the prevalence of the disorder treated by the drug.

## Methods

Eight of the 63 CDR submissions for DRDs in the CADTH article did not include a reimbursement recommendation; four were ongoing, one was withdrawn and three were Requests for Advice. The remaining 55 submissions were grouped by the prevalence of the disorder treated by the drug into three decreasing categories: <50 to >10, ≤10 to >1, and ≤1 per 100,000.

Within each prevalence category, the following were evaluated from the CDR reports:

- Time required for the CDR assessment of each drug, defined as the number of days between the submission date and the date of the final recommendation.
- Daily drug cost estimated from prices available in the CDR recommendation reports for DRDs taken daily.
- Number of randomized clinical trials (RCTs) and number of patients in the RCTs.
- Negative reimbursement recommendation rate.
- Number of CDR assessments in which (a) statistically significant evidence of clinical efficacy was demonstrated in a clinically relevant outcome in the RCT(s) included in the submission that the review committee found to be acceptable, (b) concern was expressed about the reliability of the cost-effectiveness analysis, (c) attention was drawn to the high cost of the drug or the need for a price reduction in the recommendation statement, and (d) the drug’s high cost was not mentioned in the recommendation statement but was noted elsewhere.

The CDR reports were generally transparent about the acceptability of the quality of the evidence regarding clinical efficacy and concerns about the reliability of the cost-effectiveness analysis. Attention being drawn to the high cost of a drug was derived from statements such as the cost “is exceptionally high” or treatment options exist that are “less costly,” while the need for a price reduction was identified from statements such as this drug “would not be considered cost-effective without a substantial reduction in the submitted price.”

The Kruskal-Wallis test and the chi-squared test for trend were used to analyze numeric and ordinal variables, respectively.

## Results

The prevalence rates of the disorders for which the drugs were indicated ranged widely from 0.2 to 44.7 per 100,000. None of the drugs for disorders with a prevalence of >10 per 100,000 were indicated for genetic disorders. However, nine (39.1%) and 12 (92.3%) of the drugs for disorders with prevalence rates of ≤10 to >1 and ≤1 per 100,000, respectively, were indicated for genetic disorders (a statistically significant trend, *p* = 0.002).

As the prevalence of the treated disorder decreased, the median daily cost, the negative reimbursement recommendation rate, the proportion of assessments in which concern was expressed about the reliability of the cost-effectiveness analysis, and the proportion in which attention was drawn to the high cost or the need for a price reduction significantly increased (Table 1). The median number of patients in the RCTs and the proportion of assessments with acceptable evidence of clinical efficacy decreased significantly as the prevalence decreased.

**Table 1:** CDR assessment results for 55 submissions by prevalence of treated disorder

	Prevalence of treated disorder (per 100,000)			*p*-value
	<50 to >10	≤10 to >1	≤1	
	*n* = 19	*n* = 23	*n* = 13	
Median assessment time (range)	203 (164–251)	195 (126–392)	232 (153–439)	0.409
Median daily cost (range)^a^	$55 ($13–$252)	$186 ($26–$1800)	$796 ($181–$1460)	0.0001
Median number of RCTs (range)	2 (1–6)	2 (1–4)	1 (0–4)	0.100
Median number of patients in RCTs (range)	120 (15–555)	118 (12–742)	58 (11–177)	0.020
No. with a negative recommendation	5 (26.3%)	10 (43.5%)	10 (76.9%)	0.006
No. with acceptable clinical efficacy evidence	14 (73.7%)	11 (47.8%)	2 (15.4%)	0.001
No. with concern about cost-effectiveness analysis	9 (47.4%)	18 (78.3%)	12 (92.3%)	0.004
No. with high cost or need for price reduction stated in the recommendation	3 (15.8%)	10 (43.5%)	6 (46.2%)	0.057
No. with high cost noted elsewhere in the report	0 (0.0%)	1 (4.3%)	2 (15.4%)	0.067
No. with high cost or need for price reduction noted anywhere in the report	3 (15.8%)	11 (47.8%)	8 (61.5%)	0.007

*CDR* Common Drug Review, *RCT* Randomized clinical trial

^a^Calculated for drugs used daily from prices available in the CDR reports

High cost was not a factor in submissions with a negative recommendation for any drug designed to treat a disorder with a prevalence of >10 per 100,000. However, attention was drawn to the high cost or the need for a price reduction mentioned in the CDR recommendation statement in 70.0% and 80.0% of drugs for disorders with prevalence rates of ≤10 to >1 and ≤1 per 100,000, respectively, that received a negative reimbursement recommendation (Table 2).

**Table 2:** High cost noted in 25 CDR reports with a negative reimbursement recommendation

	Prevalence of treated disorder (per 100,000)			*p*-value
	<50 to >10	≤10 to >1	≤1	
	*n* = 5	*n* = 10	*n* = 10	
No. with high cost or need for price reduction stated in the recommendation	0 (0.0%)	6 (60.0%)	5 (50.0%)	0.132
No. with high cost noted elsewhere in the report	0 (0.0%)	1 (10.0%)	3 (30.0%)	0.109
No. with high cost or need for price reduction noted anywhere in the report	0 (0.0%)	7 (70.0%)	8 (80.0%)	0.006

*CDR* Common Drug Review

As the CADTH authors noted, high cost was not a reason for any negative reimbursement recommendation from 2012 onward, whereas prior to 2012, high cost was mentioned in 85% of the negative recommendation statements for drugs for disorders with a prevalence of ≤10 per 100,000 (Table 3). Conversely, the proportion of positive reimbursement recommendation statements for drugs for disorders with a prevalence of ≤10 per 100,000 that included comments about their high cost increased from 14.3% in 2004–2011 to 100% from 2012 onward.

**Table 3:** High cost noted in the CDR reimbursement recommendation statements for drugs for disorders with a prevalence of ≤10 per 100,000, by time period

Time period	Recommendation	Prevalence of treated disorder (per 100,000)		Total
		≤10 to >1	≤1	
Before 2012	Positive	1 (20.0%)	0 (0.0%)	1 (14.3%)
	Negative	6 (85.7%)	5 (83.3%)	11 (84.6%)
2012 onward	Positive	8 (100.0%)	1 (100.0%)	9 (100.0%)
	Negative	0 (0.0%)	0 (0.0%)	0 (0.0%)

*CDR* Common Drug Review

## Discussion

Health Canada’s definition of a rare disorder as one with a prevalence of <50 per 100,000 [8] is broad, resulting in the CADTH authors including drugs for disorders with prevalence rates ranging from 0.2 to 44.7 per 100,000, i.e. the potential numbers of Canadian patients varied from 70 to 15,645. The use of this definition allowed the CADTH authors to report a negative reimbursement recommendation rate for DRDs of 45%, which is consistent with the CDR’s overall rate for all non-oncology drugs [9]. However, the aggregation of drugs for disorders with such wide prevalence rates concealed important details such as a negative recommendation rate for drugs for ultra-rare disorders, i.e. those with a prevalence of ≤1 per 100,000, of 77%.

Almost all the drugs for ultra-rare disorders were indicated for genetic disorders and had only a median number of 58 patients in the RCTs included the CDR submissions. Only 15.4% of the submissions for these drugs had acceptable evidence of clinical efficacy. One of the reasons for the high negative recommendation rate for drugs for rarer disorders is the CDR’s continuing requirement for RCTs with hard outcomes and adequate numbers to provide statistically convincing results to supply evidence of clinical efficacy. RCTs of drugs for such disorders with sufficient statistical power over a reasonable period of time are difficult, if not impossible, to perform due to the small numbers of potential participants. Moreover, when a drug becomes available for a rare disorder that severely impacts the quality or extent of life for which no effective treatment currently exists (most of the drugs for genetic disorders were unique innovative therapies), ethical considerations may prevent a RCT.

The CADTH authors categorized the reason for a negative reimbursement recommendation as insufficient or lack of evidence of clinical efficacy, lack of cost-effectiveness/high cost, or multiple issues [7]; a “lack of cost-effectiveness/high cost” was the reason in only two submissions. In the present analysis, the drug’s high cost was mentioned in the CDR recommendation statements or remarked upon in the text of the reports of 15 DRDs, all for disorders with a prevalence of ≤10 per 100,000. High cost, therefore, appears to have played a greater role in the probability of a negative CDR recommendation than the CADTH authors’ categorization suggests.

In addition, a change has occurred in how high cost DRDs are dealt with by the CDR. Before 2012, high cost was a factor in 85% of the negative reimbursement recommendations for drugs for disorders with a prevalence of ≤10 per 100,000. However, since 2012, none of the drugs in this prevalence category that received a negative recommendation had its cost noted in the recommendation statement, while 100% of drugs for disorders in this category with a positive reimbursement recommendation have included criteria advocating a price reduction or drawing attention to less expensive alternative drugs. This trend has continued in five recent CDR reports for drugs for disorders with a prevalence of ≤10 per 100,000 which all included a strong recommendation for a substantial price reduction; in one case, the CDR recommended an 80% price reduction.

This change in CDR recommendations is part of the process of integrating CADTH with the pan-Canadian Pharmaceutical Alliance (pCPA) which “conducts joint provincial/territorial negotiations for brand name drugs in Canada to achieve greater value for publicly funded drug programs and patients” [10, 11]. Representatives of the pCPA now attend CDR submission review meetings where pricing and other implementation issues that impact the pCPA and public drug plans are considered when recommendations are made [12]. The results of the present analysis of CDR recommendations for DRDs indicate that an objective of the CADTH-pCPA integration is to ensure that a negative reimbursement recommendation results in no pCPA negotiation, while a positive recommendation sets up factors for inclusion in the price negotiation―a scenario that is apparent in several recent reimbursement recommendations for drugs for common disorders.

The present analysis only included 55 CDR submissions that had a reimbursement recommendation, unlike the CADTH authors who also included the eight submissions without a recommendation in some of their analyses. The CADTH authors reported 26 submissions as having a negative recommendation, whereas 25 were identified in this analysis. Qualitative variables used in both analyses could be considered open to interpretation. However, the number of submissions with a negative reimbursement recommendation resulting from a lack of acceptable clinical efficacy evidence was similar in the CADTH analysis and the present one. A further limitation is that only small numbers were available in some comparisons, although they were usually sufficient to be able to identify statistically significant differences.

## Conclusions

The earlier aggregated analysis of submissions to the CDR for drugs for disorders with wide ranging prevalence rates concealed information of concern to patients, such as the high negative reimbursement recommendation rate for drugs for ultra-rare disorders. Since 2012, the manner in which high cost drugs for disorders with a prevalence of ≤10 in 100,000 are dealt with by the CDR has changed to harmonize its process with the system for price negotiation between public drug plans and pharmaceutical companies.

Canada remains one of few industrialized countries without an orphan drug policy to provide manufacturers with incentives to develop and market DRDs [13]. Health Canada announced an Orphan Drug Regulatory Framework in 2012 [14, 15] that is designed to increase research and innovation and facilitate patient access to DRDs, but it has yet to be approved. With many new expensive DRDs in development, a pressing need exists for Canadian federal and provincial governments to implement the Framework and to develop coherent nationwide policies to ensure that DRDs are reviewed by CADTH and funded by public drug plans in a timely and fair manner so that they are made available to all Canadians who need them.

## Abbreviations

CADTH: Canadian agency for drugs and technologies in health
CDR: Common drug review
DRDs: Drugs for rare disorders
pCPA: Pan-Canadian Pharmaceutical Alliance
RCT: Randomized clinical trial

## References

[1] Clarke JTR (2006). Is the current approach to reviewing new drugs condemning the victims of rare diseases to death? A call for a national orphan drug review policy. CMAJ.

[2] Standing Committee on Health (2007). Prescription drugs part 1 – common drug review: an F/P/T process.

[3] Drummond M, Evans B, LeLorier J, Karakiewicz P, Martin D, Tugwell P (2009). Evidence and values: requirements for public reimbursement of drugs for rare diseases – a case study in oncology. Can J Clin Pharmacol.

[4] Weeks C. Canada lags behind on rare disease drugs. Globe & Mail 2014 Feb 27. Available from: http://www.theglobeandmail.com/life/health-and-fitness/health/canada-lags-behind-on-rare-disease-drugs/article17137268. Accessed 20 Mar 2017.

[5] Feltmate K, Janiszewski PM, Gingerich S, Cloutier M (2015). Delayed access to treatments for rare diseases: who’s to blame?. Respirology.

[6] CDR Update – Issue 103. Ottawa: CADTH; 2014. Available from: https://www.cadth.ca/cdr-update-issue-103. Accessed 20 Mar 2017.

[7] Janoudi G, Amegatse W, McIntosh B, Sehgal C, Richter T (2016). Health technology assessment of drugs for rare disease: insights, trends, and reasons for negative recommendations from the CADTH common drug review. Orphanet J Rare Dis.

[8] Minister Ambrose announces patient involvement pilot for orphan drugs. Ottawa: Government of Canada; 2014. Available from: http://news.gc.ca/web/article-en.do?nid=873619. Accessed 20 Mar 2017.

[9] Rawson NSB (2015). Are the cost-effectiveness rules used by public drug plans denying coverage to Canadians with rare disorders? Canadian Health Policy.

[10] The pan-Canadian Pharmaceutical Alliance. Council of the Federation Secretariat; 2016. Available from: http://www.pmprovincesterritoires.ca/en/initiatives/358-pan-canadian-pricing-alliance. Accessed 20 Mar 2017.

[11] Rawson NSB (2016). Pan-Canadian pharmaceutical alliance: another hurdle for Canadian patients to access new drugs? Canadian Health Policy.

[12] CDR Update – Issue 119. Ottawa: CADTH; 2016. Available from: https://www.cadth.ca/cdr-update-issue-119. Accessed 20 Mar 2017.

[13] Gupta S (2012). Rare diseases: Canada’s “research orphans”. Open Med.

[14] Harper government takes action to help Canadians with rare diseases – launch of first ever Canadian framework to increase access to new treatments and information and Orphanet-Canada online portal. Ottawa: Government of Canada; 2012. Available from: http://news.gc.ca/web/article-en.do?crtr.sj1D=&crtr.mnthndVl=12&mthd=advSrch&crtr.dpt1D=&nid=698449&crtr.lc1D=&crtr.tp1D=&crtr.yrStrtVl=2012&crtr.kw=orphanet&crtr.dyStrtVl=1&crtr.aud1D=&crtr.mnthStrtVl=1&crtr.page=1&crtr.yrndVl=2012&crtr.dyndVl=30. Accessed 20 Mar 2017.

[15] Lee DK, Wong B (2014). An Orphan Drug Framework (ODF) for Canada. J Popul Ther Clin Pharmacol.

